# Supplementary material for: The Impact of Rubella Vaccine Introduction on Rubella Infection and Congenital Rubella Syndrome: A Systematic Review of Mathematical Modelling Studies
Source: Vaccines (Basel). 2021 Jan 25;9(2):84. doi: 10.3390/vaccines9020084 (PMC7912610; doi:10.3390/vaccines9020084)
Supplement: Supplementary file 1 [file vaccines-09-00084-s001.pdf]

Supplementary material

Supplement Table S1: **Search strategy for Scopus and Pubmed**

DATABASE: SCOPUS

DATE SEARCHED: 19 JUNE 2020

	Query	Results
5	(TITLE-ABS-KEY (rubella OR rubellas OR "german measles" OR "three day measles")) AND (TITLE-ABS-KEY (vaccination OR vaccin* OR immuniz* OR immunis*)) AND (TITLE-ABS-KEY (model OR models OR modelling OR modeling OR modelled OR modeled OR "theoretical stud*")) AND (LIMIT-TO (DOCTYPE , "ar") OR LIMIT-TO (DOCTYPE , "re") OR LIMIT-TO (DOCTYPE , "cp"))	946 document results
4	(TITLE-ABS-KEY (rubella OR rubellas OR "german measles" OR "three day measles")) AND (TITLE-ABS-KEY (vaccination OR vaccin* OR immuniz* OR immunis*)) AND (TITLE-ABS-KEY (model OR models OR modelling OR modeling OR modelled OR modeled OR "theoretical stud*"))	987 document results
3	TITLE-ABS-KEY (model OR models OR modelling OR modeling OR modelled OR modeled OR "theoretical stud*")	13,477,036 document results
2	TITLE-ABS-KEY (vaccination OR vaccin* OR immuniz* OR immunis*)	620,481 document results
1	TITLE-ABS-KEY (rubella OR rubellas OR "german measles" OR "three day measles")	24,817 document results

DATABASE: PUBMED

DATE SEARCHED: 19 JUNE 2020

	Query	Results
#4	Search: #1 AND #2 AND #3	447 document results
#3	Search: Models, Theoretical [mh] OR model*[tiab] OR theoretical stud*[tiab]	3,807,970 document results
#2	Search: Vaccination[mh] OR vaccin*[tiab] OR immuniz*[tiab] OR immunis*[tiab]	402,546 document results
#1	Search: Rubella[mh] OR rubella*[tiab] OR german measles[tiab] OR three day measles[tiab]	14,410 document results

Supplement Table S2: Risk of bias tool for assessment of included studies

	Criterion (adapted from Fone *et al.* & Caro *et al.)*	Considerations (adapted from Fone et al. and Caro et al.)	Score considerations (0, poor to 2, good)	
1	**Are the aims and objectives clear?**	Are the research questions and modelling objectives clearly defined?	0 Not stated<br>1 Stated but vague<br>2 Stated and focussed	Definitions: max 8 points
2	**Is the setting and population clearly defined?**	Does the paper clearly state the setting (e.g. geographical location, high/low TB burden)?	0 Not stated<br>1 Stated but vague or details missing<br>2 Stated and focussed	
		In health economics models, has the perspective been stated?		
		Does the paper clearly state the modelled population? (e.g. patient or population group characteristics)		
		Have sub-populations necessary for the research question and setting been modelled?		
3	**Are the intervention and comparators adequately defined?**	Does the paper clearly state the population(s) targeted for vaccination?	0 Not stated or very unclear<br>1 Stated but details missing<br>2 Stated and all necessary details stated	
		Does the paper clearly define the vaccine characteristics (e.g. vaccine efficacy, duration of protection, number of doses, waning, timing)?		
		If there is a comparator (no vaccine, baseline or alternative intervention scenario), is it clearly defined?		
4	**Are the outcome measures defined and answer the research question?**	Does the paper clearly define the outcomes of interest?	0 Not stated, very unclear or not suited to research question<br>1 Stated but details missing or not directly aligned with research question<br>2 Stated, all necessary details stated, and aligned with research question	
		Do the outcomes correspond to the research question?		
5	**Are the model structure and time horizon clearly described and**	Is the model structure clearly reported and appropriate for the research question?	0 Not appropriate model structure, or poor/no description of model<br>1 Incomplete description, and/or appropriate in part for research question	Model methods: max 4 points
		Does the model reflect current knowledge of disease natural history?		

	**appropriate for the research question?**	Is the time horizon and time step of the model clearly stated and appropriate to the research question (i.e. is it long enough to capture health effects)?	2 Complete and reproducible, appropriate structure and time horizon	
6	**Are the modelling methods appropriate for the research question and adequately described?**	Were the modelling methods clearly described, and suited to the research question?	0 Not appropriate model structure, or poor/no description of methods<br>1 Incomplete description, and/or appropriate in part for research question<br>2 Complete and reproducible, appropriate method	
7	**Are the parameters, ranges and data sources specified?**	Are all parameters and their ranges reported?<br>Are the data sources for parameters reported?	0 Poorly reported<br>1 Some information missing<br>2 Complete reporting of parameters, ranges and data sources	Model inputs: max 6 points
8	**Are any assumptions explicit and justified?**	Are all assumptions explicit and justified?	0 Not reported<br>1 Explicit<br>2 Explicit and justified	
9	**Is the quality of data considered and is uncertainty explored through uncertainty and/or sensitivity analyses?**	Are data limitations discussed? Are any of the sources known to the reviewer to be inappropriate (e.g. do not match the parameter, are outdated, or known to be poor quality)?<br>Is uncertainty in model structure, parameters and/or assumptions explored through uncertainty and/or sensitivity analyses?	0 No sources or uncertainty<br>1 Partially addressed, and/or some data inappropriate<br>2 Fully addressed	
10	**Is the method of fitting described and suitable?**	Is the method of fitting/calibrating the model clearly described?<br>Is the method of model fitting/calibration suitable?	0 Not done, unsuitable method or poor/no description<br>1 Incomplete description or method not optimal<br>2 Complete description and suitable methods	Fitting/ validation: max 4 points
11	**Has the model been validated?**	Has an assessment of validity of the results been made by comparing across one or more different model structures, or against a validation data set?	0 Not considered<br>1 States criteria for validation<br>2 Validation undertaken	
12	**Have the results been clearly and completely presented, with a range of uncertainty?**	Have the outcome values and their uncertainty ranges for each intervention/scenario been reported?<br>Do the results match the objectives?<br>Are sensitivity analyses clearly reported?	0 Not reported, very unclear or not suited to research question<br>1 Stated, but ranges or planned sensitivity analyses missing and/or not directly aligned with research question	Results: max 4 points

			2 Values and ranges and planned sensitivity analyses reported and aligned with research question.	
13	**Are the results appropriately interpreted and discussed in context?**	Does the discussion reflect a fair and balanced interpretation of the results?	0 No/poor discussion<br>1 Some discussion but key points, limitations or context missed<br>2 Full discussion of key points in context, generalisability considered, limitations discussed	
		Are the results of the study discussed in context and is generalisability considered?		
		Are possible biases and limitations discussed?		
14	**Are the funding source and conflicts of interest reported?**	Is the funding and the role of the funder clearly stated?	0 No statement of funding or conflicts<br>1 Funding or conflicts reported<br>2 Funding and conflict statement	Conflicts: Max 2 points

Overall Scoring: Max 28 points	
Very high	>22
High	19-22
Medium	14-18
Low	<14

Supplement Table S3: PRISMA checklist

Section/topic	#	Checklist item	Reported on page #
**TITLE**			
Title	1	Identify the report as a systematic review, meta-analysis, or both.	1
**ABSTRACT**			
Structured summary	2	Provide a structured summary including, as applicable: background; objectives; data sources; study eligibility criteria, participants, and interventions; study appraisal and synthesis methods; results; limitations; conclusions and implications of key findings; systematic review registration number.	2
**INTRODUCTION**			
Rationale	3	Describe the rationale for the review in the context of what is already known.	4
Objectives	4	Provide an explicit statement of questions being addressed with reference to participants, interventions, comparisons, outcomes, and study design (PICOS).	4
**METHODS**			
Protocol and registration	5	Indicate if a review protocol exists, if and where it can be accessed (e.g., Web address), and, if available, provide registration information including registration number.	6
Eligibility criteria	6	Specify study characteristics (e.g., PICOS, length of follow-up) and report characteristics (e.g., years considered, language, publication status) used as criteria for eligibility, giving rationale.	5
Information sources	7	Describe all information sources (e.g., databases with dates of coverage, contact with study authors to identify additional studies) in the search and date last searched.	5
Search	8	Present full electronic search strategy for at least one database, including any limits used, such that it could be repeated.	S1
Study selection	9	State the process for selecting studies (i.e., screening, eligibility, included in systematic review, and, if applicable, included in the meta-analysis).	6
Data collection process	10	Describe method of data extraction from reports (e.g., piloted forms, independently, in duplicate) and any processes for obtaining and confirming data from investigators.	5
Data items	11	List and define all variables for which data were sought (e.g., PICOS, funding sources) and any assumptions and simplifications made.	5
Risk of bias in individual studies	12	Describe methods used for assessing risk of bias of individual studies (including specification of whether this was done at the study or outcome level), and how this information is to be used in any data synthesis.	S2
Summary measures	13	State the principal summary measures (e.g., risk ratio, difference in means).	5

Synthesis of results	14	Describe the methods of handling data and combining results of studies, if done, including measures of consistency (e.g., I^2) for each meta-analysis.	6

Section/topic	#	Checklist item	Reported on page #
Risk of bias across studies	15	Specify any assessment of risk of bias that may affect the cumulative evidence (e.g., publication bias, selective reporting within studies).	5
Additional analyses	16	Describe methods of additional analyses (e.g., sensitivity or subgroup analyses, meta-regression), if done, indicating which were pre-specified.	6
**RESULTS**			
Study selection	17	Give numbers of studies screened, assessed for eligibility, and included in the review, with reasons for exclusions at each stage, ideally with a flow diagram.	6
Study characteristics	18	For each study, present characteristics for which data were extracted (e.g., study size, PICOS, follow-up period) and provide the citations.	6 & 7
Risk of bias within studies	19	Present data on risk of bias of each study and, if available, any outcome level assessment (see item 12).	18
Results of individual studies	20	For all outcomes considered (benefits or harms), present, for each study: (a) simple summary data for each intervention group (b) effect estimates and confidence intervals, ideally with a forest plot.	16 & 17
Synthesis of results	21	Present the main results of the review. If meta-analyses are done, include for each, confidence intervals and measures of consistency.	7 & 8
Risk of bias across studies	22	Present results of any assessment of risk of bias across studies (see Item 15).	18
Additional analysis	23	Give results of additional analyses, if done (e.g., sensitivity or subgroup analyses, meta-regression [see Item 16]).	N/A
**DISCUSSION**			
Summary of evidence	24	Summarize the main findings including the strength of evidence for each main outcome; consider their relevance to key groups (e.g., healthcare providers, users, and policy makers).	9
Limitations	25	Discuss limitations at study and outcome level (e.g., risk of bias), and at review-level (e.g., incomplete retrieval of identified research, reporting bias).	10
Conclusions	26	Provide a general interpretation of the results in the context of other evidence, and implications for future research.	10
**FUNDING**			

Funding	27	Describe sources of funding for the systematic review and other support (e.g., supply of data); role of funders for the systematic review.	10

Supplement Table S4: Characteristics of excluded studies

Unique ID of study (First author surname, year of publication & first word of title)	Is this a mathematical modelling study?	Does the study use a dynamic transmission model?	Does the study simulate rubella vaccine introduction?	Did the study compare vaccine introduction	Did study report time (in years) to the elimination of CRS?	Did study report time (in years) to rubella elimination?	Did study describe trends in rubella or CRS incidence?	Did study report number vaccinated per CRS case averted?	Did study report cost of vaccine introduction	What is the time horizon for the simulations in years?	Reason for exclusion
Hincapie-Palacio_2010_Simulating	Yes	Yes	Yes	Yes	No	No	No	No	No	2	Ineligible outcomes
Reinert_2003_Evaluation	Yes	No	Yes	Yes	No	No	No	No	No	30	Ineligible design
Beraud_2018_Resurgence	Yes	No	No	No	No	No	No	No	No	2	Ineligible design
Buonomo_2011_A Simple	Yes	Yes	Yes	Yes	No	No	Yes	No	No	3	Ineligible intervention duration
Edmunds_2000_Modelling	Yes	Yes	No	Yes	No	No	Yes	No	No	40	Ineligible intervention
Edmunds_2000_The pre-vaccination	Yes	Yes	No	No	No	No	No	No	No	N/A	Ineligible intervention
Feng_2020_Influence	Yes	Yes	No	No	No	No	No	No	No	N/A	Ineligible intervention
Gao_2013_Models	Yes	Yes	No	No	Yes	Yes	Yes	No	No	60	Ineligible intervention
Hachiya_2018_Evaluation	Yes	No	No	No	No	No	No	No	No	N/A	Ineligible design
Lessler_2013_Balancing	Yes	No	Yes	Yes	No	No	No	No	No	N/A	Ineligible design
Metcalf_2011_Rubella	Yes	Yes	No	N/A	No	No	No	No	No	N/A	Ineligible intervention
Metcalf_2012_Impact	Yes	Yes	Yes	Yes	Yes	No	Yes	No	No	30	Ineligible setting
Metcalf_2012_Structured	Yes	Yes	No	No	No	No	Yes	No	No	35	Ineligible intervention
Thompson_2016_Synthesis	No	No	No	No	No	No	No	No	No	N/A	Ineligible design
Thompson_2016_The Cost	No	No	No	No	No	No	No	No	No	N/A	Ineligible design
Thompson_2017_Modelling	Yes	Yes	Yes	No	No	No	No	No	No	N/A	Ineligible intervention

Vynnycky_2019_The_impact	Yes	Yes	Yes	No	Yes	No	Yes	No	Yes	30	Ineligible intervention

Supplement Table S5: Risk of bias assessment of included studies

Study	Risk of bias item														Final score	Quality Grading
	Aim & objectives	Setting & population	intervention & comparators	Outcome measures	Model structure & time horizon	Modelling methods	Parameters, ranges & data sources	Assumptions	Quality of data & exploration of uncertainty	Method of fitting	Model validation	Results	Interpretation & discussion of results	Funding sources & conflicts of interest		
**Gao (2006)**	2	2	2	2	2	2	1	2	1	0	0	1	2	0	19	**High**
**Jazbec (2004)**	2	2	2	2	2	2	1	2	1	0	2	1	1	0	20	**High**
**Motaze (2020)**	2	2	2	2	2	2	0	2	2	0	0	2	2	2	22	**High**
**Vynnycky (2016)**	2	2	2	2	2	2	0	2	2	0	0	2	2	2	22	**High**
**Wesolowski (2016)**	2	2	2	2	2	2	0	2	1	0	0	2	1	2	20	**High**
**Winter (2017)**	2	2	2	2	2	2	0	2	2	0	0	2	2	2	22	**High**

**Wu (2016)**	2	2	2	2	2	2	2	2	2	0	0	2	2	2	24	**Very high**
